# Impact of Technical Standardization on Pneumothorax and Chest Tube Insertion Rates: A Retrospective Learning Curve Analysis of CT-Guided Lung Biopsies

**DOI:** 10.3390/jcm14144838

**Published:** 2025-07-08

**Authors:** Rosa Alba Pugliesi, Younesse Nasser, Amina Benchekroun, Roua BenAyed, Andreas H. Mahnken, Nour Maalouf, Jonas Apitzsch

**Affiliations:** 1 Section of Radiology—Department of Biomedicine, Neuroscience and Advanced Diagnostics (BiND), University of Palermo, Via del Vespro 129, 90127 Palermo, Italy; 2 Department of Radiology and Nuclear Medicine, Helios Hospital Pforzheim, 75175 Pforzheim, Germany; younesnasser84@gmail.com (Y.N.); amina.benchekroun@hotmail.com (A.B.); roua.benayed@helios-gesundheit.de (R.B.); jonas.apitzsch@helios-gesundheit.de (J.A.); 3 Department of Diagnostic and Interventional Radiology, University Hospital of Marburg, 35043 Marburg, Germany; andreas.mahnken@staff.uni-marburg.de; 4 Department of Radiology and Nuclear Medicine, Tϋbingen University Hospital, 72076 Tϋbingen, Germany; nourmaalouff@gmail.com

**Keywords:** CT-guided lung biopsy, pneumothorax, needle angulation, procedural knowledge, chest tube insertion

## Abstract

**Background:** Pneumothorax (PTX) is the most common complication of CT-guided lung biopsies. New technical advances, namely the optimization of needle approach angles within an a priori defined “safe zone,” are intended to reduce this risk. This study evaluates whether PTX incidence and chest tube placement decreased significantly after these technical advances were implemented. **Methods**: We retrospectively analyzed 118 consecutive patients who had undergone CT-guided lung biopsy between 9 January 2020, and 4 April 2025. The study was divided into three periods of increasingly growing institutional procedural experience: Pre-Knowledge (January 2020–March 2022; n = 45), Partial Knowledge (April–December 2022; n = 18), and Full Knowledge (January 2023–April 2025; n = 55). PTX incidence and chest tube use were compared across periods using chi-square and Fisher’s exact tests, while Kaplan–Meier survival analysis was used to evaluate PTX-free survival over time. **Results:** Overall PTX incidence significantly declined from 71.1% in the Pre-Knowledge Period to 21.8% in the Full Knowledge Period (*p* < 0.000001). Rates of chest tube placements also decreased from 17.8% to 9.1%, although this difference was not statistically significant (*p* = 0.372). Kaplan–Meier analysis showed a statistically significant improvement in PTX-free survival over time (indicating improvement in the timing of complication onset; *p* = 0.0042). Procedural optimization was also fostered by a large median intrapulmonary needle length and consistent needle angulation within the safe zone. **Conclusions:** Formal implementation of needle angle optimization and procedural protocol standardization has effectively reduced the frequency and severity of PTX following CT-guided lung biopsies. These results highlight the benefit of continuous education and technique standardization in improving patient safety and clinical outcomes.

## 1. Introduction

Image-guided lung biopsy plays an essential role in the diagnosis and treatment planning of pulmonary nodules by providing tissue samples for pathological analysis [1]. While widely used, the technique is not to be taken lightly due to the risk for complications, most commonly PTX [2]. PTX is clinically significant since it can necessitate chest tube insertion and, on occasion, lead to prolonged hospitalization or procedural failure [3,4].

The increasing use of tissue-based molecular profiling and targeted therapy has created a growing demand for percutaneous lung biopsies in recent years [5,6]. However, PTX rates greater than 25% have been reported in large patient populations during or shortly after computed tomography (CT)-guided transthoracic lung biopsies [7]. In addition to posing a direct threat, PTX can reduce diagnostic yield and complicate patient management [8].

Technical variables, particularly the trajectory of the biopsy needle in relation to the pleura, have been shown to influence the risk of PTX [9]. Our prior retrospective analysis identified the pleura–needle angle as a significant predictor of PTX: patients who developed PTX had a mean angle of 74.0°, whereas unaffected patients had a mean angle of 94.7° (*p* = 0.028). These findings prompted us to adjust our technique by selecting needle tracks closer to perpendicular (≈90°) to minimize pleural injury and reduce air leakage [10]. 

A subsequent study by our group [11] operationally defined a procedural “safe zone” by demonstrating that pleura–needle angles between 80° and 100° were associated with a significantly lower rate of PTX (30.0%) compared with angles greater than 10° from perpendicular (71.8%; *p* = 0.0023), leading to the formal implementation of this angular range in routine clinical practice.

The present study builds upon previous work and investigates whether the formal implementation of technical refinements, specifically needle angle optimization within the safe zone, led to a statistically significant reduction in PTX rates and chest tube insertion rates over time. This study aims to assess whether the formal implementation of needle angle optimization led to a measurable reduction in PTX rates and improved clinical outcomes.

## 2. Materials and Methods

A retrospective analysis was performed in 118 consecutive patients (66 males, 52 females; median age: 69 years; age range: 49–90 years) who underwent CT-guided lung biopsy between 9 January 2020, and 4 April 2025. All procedures were performed exclusively by a single interventional radiologist with 18 years of experience in interventional radiology, thereby eliminating operator-dependent variability. Patients with pre-existing lung conditions predisposing to PTX, such as emphysema and cystic lung disease, were included and classified under the COPD (Chronic Obstructive Pulmonary Disease) variable.

The study interval was divided into three periods based on the progressive implementation and institutional dissemination of procedural expertise: the Pre-Knowledge Period (9 January 2020–31 March 2022), the Partial Knowledge Period (1 April 2022–31 December 2022), and the Full Knowledge Period (1 January 2023–4 April 2025). The analysis focused on the distribution and proportion of PTX subtypes, as well as the overall incidence of PTX, across these three defined procedural knowledge periods. 

This stratification was guided by the increasing institutional uptake and refinement of standardized, evidence-based biopsy protocols to reduce complication rates.

In the Pre-Knowledge phase, procedures were performed without unified guidelines, whereas the Partial Knowledge phase marked the transition toward protocol implementation. In the Full Knowledge phase, procedural techniques were consistently optimized and uniformly applied. The classification reflects institutional procedural maturity rather than individual operator learning, as all biopsies were performed by the same experienced radiologist. The incidence of PTX and the need for chest drainage were documented in all patients. In all cases, only a single pleural puncture was made, with no multiple needle passages through the pleura, further standardizing the procedure. The primary endpoint was the incidence of PTX, classified into three clinically relevant categories. PTX was categorized into three types for analysis:

(1) Transient intraprocedural PTX that began during needle passage but had resolved by the end of the procedure.
(2) Persistent, clinically inapparent PTX that did not spontaneously resolve but remained small and needed no treatment.
(3) Clinically significant PTX that needed therapy by chest tube insertion. Baseline characteristics are detailed in Table 1.

Classification as clinically significant PTX was based on symptomatic and radiological findings suggesting progression, not all of which required immediate chest tube placement.

PTX was assessed using an immediate post-biopsy CT, and a follow-up CT within 24 h was performed only when clinically indicated based on symptoms or chest X-ray findings. Routine monitoring otherwise relied on symptom-based observation and chest radiographs. All patients provided informed consent more than 24 h prior to the intervention. Ethical approval was given by the local ethics committee (F-2021-038).

### 2.1. Inclusion and Exclusion Criteria

Inclusion criteria were undergoing CT-guided lung biopsy during the study interval and having a lesion suitable for biopsy based on its size, location, and proximity to the pleura. Exclusion criteria included lesions smaller than 4 mm in diameter, an International Normalized Ratio (INR) > 1.5, or inability to comply with procedural requirements.

### 2.2. Biopsy Procedure

All CT-guided lung biopsies were performed using a semi-automatic TruCut 18G needle (Möller Medical GmbH, Fulda, Germany) and a 17G trocar (Möller Medical GmbH, Fulda, Germany). Biopsies were performed in supine or prone positions based on lesion location.

Local anesthesia (mepivacaine 1%) was used and a small skin incision was made to enable the coaxial needle to be placed at the edge of the lesion. A CT scan of the intended biopsy area was performed at end-expiration breath-hold using a SOMATOM Definition Edge CT scanner (Siemens Healthineers, Forchheim, Germany) with settings of of 20 mA and 120 kV. Fixation in formaldehyde and histopathological examination were performed on the biopsy samples post-biopsy.

### 2.3. Statistical Analysis

The primary outcome—incidence of PTX—was compared across the three procedural knowledge periods using Pearson’s chi-square test to analyze the difference in PTX versus non-PTX events.

PTX-free survival probabilities were also investigated via Kaplan–Meier survival curves, with the log-rank test employed to compare groups. All statistical analyses were conducted using a significance level of 0.05. Data analysis was performed using R software (version 4.4.2; R Foundation for Statistical Computing, Vienna, Austria).

## 3. Results

A total of 59 PTX events occurred among the 118 patients, with marked variation across the three procedural-knowledge periods.

Table 2 summarizes the distribution of PTX subtypes, overall incidence, chest-tube insertions, and each period’s contribution to the overall chi-square statistic. While the Partial Knowledge Period showed the highest relative PTX frequency (83.3%), the Pre-Knowledge Period included the highest absolute PTX count (32, 71.1%). To statistically evaluate deviations from expected PTX occurrence, observed (O) and expected (E) PTX counts were compa red using chi-square contributions ((O−E)^2^/E). The highest chi-square contribution occurred in the Partial Knowledge Period (2.520), reflecting a PTX frequency greater than expected. The Full Knowledge Period contributed 2.077 to the chi-square statistic—reflecting fewer PTX cases than expected—whereas the Pre-Knowledge Period’s contribution was negligible (0.001), indicating that its observed PTX count closely matched the expected value. The overall chi-square test was significant (χ^2^ = 33.495, df = 2, *p* < 0.000001), suggesting a very high correlation between level of knowledge and PTX occurrence.

Table 2 illustrates chest tube insertion data, which reveals that although the incidence of clinically significant PTX (those requiring drainage) decreased across periods, this reduction was not statistically significant (Fisher’s exact test *p* = 0.372). This supports the observation that, although notably decreased PTX rates were observed, clinical severity warranting intervention improved only numerically.

A subgroup analysis of patients with COPD (n = 40) versus those without COPD (n = 78) showed PTX in 10 (25.0%) and 22 (28.2%) patients, respectively (χ^2^ = 0.02, *p* = 0.88), indicating no statistically significant difference in PTX incidence between groups.

**Figure 1:**
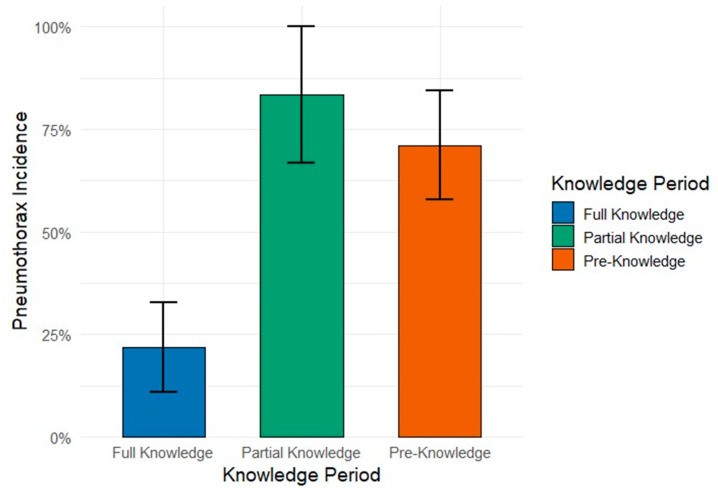
PTX incidence by procedural knowledge period. Although the Partial Knowledge Period showed the highest proportional incidence (83.3%), the Pre-Knowledge Period had the highest absolute count. A chi-square test indicated a significant association (*p* < 0.000001).

Figure 1 illustrates these differences visually, highlighting procedural improvements over time.

To evaluate whether PTX severity varied by knowledge period, Fisher’s exact test was used to compare rates of clinically significant PTX, defined as cases requiring chest tube insertion (Table 3).

No statistically significant difference was observed (*p* = 0.372), which suggests that, despite falling PTX rates, the clinical severity of events requiring drainage was not significantly modified.

**Figure 2:**
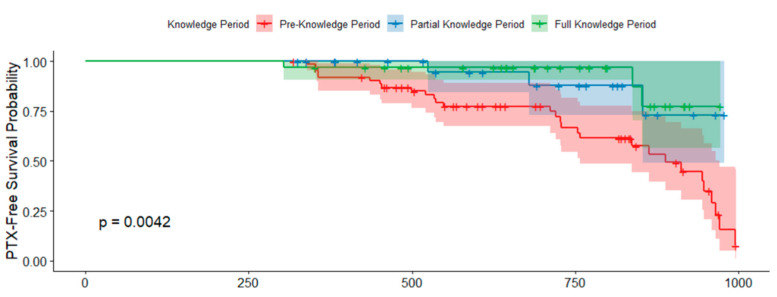
Kaplan–Meier survival curves illustrating PTX-free survival (time to first PTX event) across procedural knowledge periods.

In addition, Kaplan–Meier survival analysis demonstrated a significant difference in PTX-free survival across the knowledge periods (*p* = 0.0042), once again validating the beneficial impact of increased technical proficiency on patient outcomes over time. Figure 2 illustrates these findings, where the survival plots for Pre-Knowledge, Partial Knowledge, and Full Knowledge Periods are depicted in red, blue, and green, respectively. There was a significant improvement in PTX-free survival with increasing experience. 

**Figure 3:**
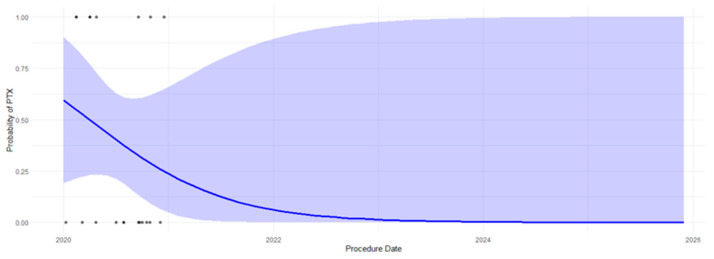
Logistic regression analysis of the association between procedure date and PTX occurrence. Each blue point represents a single observation, with the *y*-axis showing PTX occurrence (1 = Yes, 0 = No) and the *x*-axis representing the date of the CT-guided lung biopsy. Shaded areas denote the 95% confidence interval of the fitted model. The blue curve indicates the logistic regression fit, modeling the declining PTX risk over time (*p* < 0.0001).

To further assess the temporal trend in PTX occurrence, Figure 3 illustrates a logistic regression model depicting the relationship between procedure date and PTX occurrence. The analysis is consistent with a declining probability of PTX over time with a significant temporal trend (*p* < 0.0001), showing that the gradual increase in knowledge-based enhancements resulted in a decline in PTX prevalence.

### 3.1. Lesion Size Analysis

Lesion size was recorded in millimeters for all cases and ranged from 2.6 mm to 115 mm. The median lesion size did not differ significantly across the three procedural knowledge periods (Pre-Knowledge, Partial Knowledge, and Full Knowledge). Statistical comparison among the three groups using the Kruskal–Wallis test also confirmed no significant difference (*p* = 0.48), indicating a comparable distribution of lesion sizes over time. This finding suggests that changes in PTX incidence were not confounded by variations in lesion size.

### 3.2. Lesion Location Distribution

Lesion locations were initially documented by segment and then grouped into five standard lung lobes for uniform analysis: right upper lobe (RUL), right middle lobe (RML), right lower lobe (RLL), left upper lobe (LUL), and left lower lobe (LLL). Lesions spanning multiple segments were graded in the respective lobes accordingly. Lesion-site distribution did not differ significantly among the three knowledge periods (chi-square *p* = 0.72). This validates that anatomical site did not disrupt the observed improvement in PTX outcomes.

### 3.3. Intrapulmonary Needle Length Analysis

**Figure 4:**
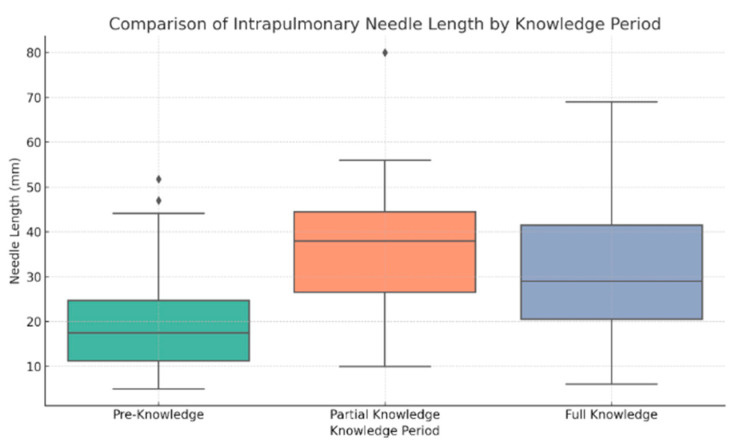
Boxplot comparison of intrapulmonary needle lengths across procedural knowledge periods. The Pre-Knowledge Period showed shorter and more variable needle depths. The Partial Knowledge Period had the longest median depth, while the Full Knowledge Period demonstrated a more standardized and intermediate range, likely reflecting procedural optimization. Black dots indicate individual cases.

In contrast to lesion characteristics, intrapulmonary needle length varied significantly across knowledge periods (Kruskal–Wallis *p* = 0.00019). Median needle lengths were lowest during the Pre-Knowledge Period (17.5 mm; IQR: 11.3–24.8 mm), highest during the Partial Knowledge Period (38.0 mm; IQR: 26.5–44.5 mm), and moderate during the Full Knowledge Period (29.0 mm; IQR: 20.5–41.5 mm). Post-hoc Bonferroni-corrected Mann–Whitney U tests showed significant differences between Pre- and Full-Knowledge periods (adjusted *p* < 0.000001) and between Pre- and Partial-Knowledge periods (adjusted *p* = 0.000004), whereas the Partial- and Full-Knowledge periods did not differ (adjusted *p* = 1.00) (Figure 4). These results indicate a trend toward more ideal and uniform needle paths over time, which may be contributing to decreasing rates of PTX.

## 4. Discussion

This retrospective study investigated if the rates of PTX and chest tube insertion after CT-guided lung biopsies reduced over time with the accumulation of procedural volume and technical refinement, i.e., ideal needle orientation [12,13]. Statistically significant reductions in PTX rates were observed across the Pre-Knowledge, Partial Knowledge, and Full Knowledge Periods (*p* < 0.000001), thus confirming the working hypothesis that modifications to technique, with emphasis on optimization and standardization of angulation, have a major clinical impact. Post hoc comparisons indicated that the large difference was due to the Full Knowledge Period relative to both the Pre-Knowledge (adjusted *p* < 0.000001) and Partial Knowledge (adjusted *p* = 0.0003) Periods, whereas the Pre and Partial Knowledge groups did not differ (adjusted *p* = 0.42). Hence, the improvement occurred primarily in the Full Knowledge Period rather than progressively over time.

PTX incidence actually peaked in the Partial Knowledge Period (83.3%) and then fell sharply in the Full Knowledge Period (21.8%), underscoring the benefit of full protocol implementation.

Although chest-tube insertion also declined in the Full-Knowledge period (9.1% vs 17.8%), the difference was not statistically significant (*p* = 0.372) and should therefore be interpreted cautiously; nonetheless, the numerical reduction suggests a potential decrease in complication severity that supports the benefit of procedural refinement. These findings underscore the need for larger prospective trials to determine if such gains result in statistically significant clinical benefits.

Despite COPD being a known PTX risk factor, subgroup analysis showed comparable PTX rates in patients with COPD (25.0%) and without COPD (28.2%; *p* = 0.88), implying that standardized needle angulation and operator expertise may mitigate underlying respiratory risk and make the protocol broadly applicable. This was substantiated by Kaplan–Meier survival analysis, in which PTX-free survival was considerably improved over time (*p* = 0.0042), indicating that the time to PTX occurrence was longer in subsequent procedural time intervals. This suggests that the protocol enhancements were effective in delaying or potentially preventing the onset of PTX. Because the log-rank test compares event timing, its significant result here reinforces that patients in the Full-Knowledge period experienced substantially longer PTX-free intervals than those in earlier periods. These findings are consistent with previous studies highlighting the importance of 80° to 100° needle–pleura angulation in the “safe zone” to reduce pleural trauma and hence PTX risk [11,14]. In particular, when PTXs did occur despite optimal technique, they were smaller, less clinically significant, and necessitated procedures such as chest drainage less frequently. While the log-rank analysis does not provide direct information about disease severity, the overall reduction in clinically significant PTX, albeit not statistically significant, reinforces the procedural benefits. These data underscore the clinical value of technique standardization and consistent angulation awareness. 

The difference between *p*-values for log-rank and chi-square tests indicates a useful statistical nuance: the chi-square test assesses differences in overall PTX occurrence, whereas the log-rank test assesses differences in timing and pattern of PTX occurrence. Together, the analyses confirm that improved technique not only correlates with fewer complications but also with more favorable onset patterns, leading to less severe disease courses. These results align with biomechanical reasoning, which contends that more perpendicular needle punctures cause less damage to the tissue and lower the risk of large or clinically significant PTX [10,11].

These results have major clinical consequences: PTX following refined procedures is increasingly a minor instead of a major complication. However, several limitations should be considered. First, the retrospective design introduces potential selection and reporting biases. Second, the relatively small sample sizes, especially when divided into three subgroups, may limit statistical power and generalizability. Third, confounding factors such as lesion type, operator variability beyond the single operator studied, and patient-specific respiratory mechanics were not fully controlled and may have influenced the outcomes.

Notwithstanding these limitations, our findings support procedural standardization and targeted knowledge transfer as effective strategies for lowering PTX incidence and severity during CT-guided lung biopsy [15,16,17]. By validating the clinical advantages of needle angulation awareness and promoting ongoing education and quality improvement, our findings support enhanced patient safety and procedural efficiency [18,19].

Future prospective multicenter trials involving larger cohorts and controlled operator variability are warranted to confirm these results and examine more widespread implementation strategies. Particular attention should be paid to correlating needle angle with clinical outcomes including hospitalization duration, chest tube dependency, and recovery trajectories. Additional research might also clarify whether PTX can increasingly be managed as a minor complication, potentially reducing patient morbidity and healthcare costs [20,21,22,23].

## 5. Conclusions

This study demonstrates that formalized procedural knowledge application and optimal needle angulation reduce both the incidence and the clinical effect of PTX following CT-guided lung biopsies. The observed decreased rates of chest tube placements, though not statistically significant, suggest that better technique can also mitigate the clinical effects of complications. These findings suggest standardized training, needle angulation optimization, and continued procedural optimization as cornerstones of best practice in thoracic interventional radiology to ensure maximum procedural success and patient safety.

## Figures and Tables

**Table 1:** Baseline demographic, clinical, and procedural characteristics of the patients undergoing CT-guided lung biopsy. Age, sex, COPD prevalence, PTX types, lesion distribution by lung lobe, and procedural parameters including lesion size and needle depth are detailed.

Characteristic	Value (n, %)
Total Patients Analyzed	118
Mean Age (years)	69 (median, range 49–90)
Sex, Male/Female	66 (55.9%)/52 (44.1%)
COPD, n (%)	40 (33.9%)
PTX Types, n (%)	
Transient PTX	28 (23.7%)
Persistent Inapparent PTX	7 (5.9%)
Clinically Significant PTX	24 (20.3%)
Lobe Location of Lesions, n (%)	
Right Upper Lobe (RUL)	38 (32.2%)
Right Middle Lobe (RML)	15 (12.7%)
Right Lower Lobe (RLL)	35 (29.7%)
Left Upper Lobe (LUL)	16 (13.6%)
Left Lower Lobe (LLL)	14 (11.9%)
Lesion Size (mm), Mean ± SD	38.2 ± 25.8
Needle Depth (mm), Mean ± SD	28.4 ± 20.1

**Table 2:** Summary of pneumothorax (PTX) subtypes, chest tube insertion rates, and chi-square contributions across procedural knowledge periods. O = observed PTX; E = expected PTX; PTX = pneumothorax.

Knowledge Period	Total Patients (n)	Transient PTX n (%)	Persistent Inapparent PTX n (%)	Clinically Significant PTX n (%)	Total PTX n (%)	No PTX n (%)	Chest Tube n (%)	Observed PTX (O)	Expected PTX (E)	Chi-Square Contribution ((O−E)^2^/E)
Pre-Knowledge	45	15 (33.3%)	4 (8.9%)	13 (28.9%)	32 (71.1%)	13 (28.9%)	8 (17.8%)	32	31.87	0.001
Partial Knowledge	18	6 (33.3%)	3 (16.7%)	6 (33.3%)	15 (83.3%)	3 (16.7%)	1 (5.6%)	15	9.98	2.520
Full Knowledge	55	7 (12.7%)	0 (0.0%)	5 (9.1%)	12 (21.8%)	43 (78.2%)	5 (9.1%)	12	18.15	2.077
Total	118	28 (23.7%)	7 (5.9%)	24 (20.3%)	59 (50.0%)	59 (50.0%)	14 (11.9%)	59	59.99	4.598

**Table 3:** Chest tube insertions across knowledge periods.

Knowledge Period	Chest Tube (Yes)	No Chest Tube	Total Patients
Pre-Knowledge	8 (17.8%)	37 (82.2%)	45
Partial Knowledge	1 (5.6%)	17 (94.4%)	18
Full Knowledge	5 (9.1%)	50 (90.9%)	55
Total	14 (11.9%)	104 (88.1%)	118

Fisher’s Exact Test: *p* = 0.372.

## Data Availability

The data presented in this study are openly available in FigShare at Pugliesi, Rosa Alba; Christoph Apitzsch, Jonas (2025). Dataset study—The Impact of Technical Standardization on Pneumothorax and Chest Tube Insertion Rates: A Retrospective Learning Curve Analysis of CT-Guided Lung Biopsies. figshare. Dataset. https://doi.org/10.6084/m9.figshare.28925330.
